# College on the Margins: A Comprehensive Case Study of Three College-in-Prison Programs in the Southern United States

**DOI:** 10.3390/bs15101351

**Published:** 2025-10-02

**Authors:** Haruna Suzuki, John C. Begeny

**Affiliations:** 1 Department of Educational Leadership, Policy, and Human Development, College of Education, North Carolina State University, Raleigh, NC 27606, USA; hsuzuki12@yahoo.com; 2 Department of Psychology, College of Humanities and Social Sciences, North Carolina State University, Raleigh, NC 27606, USA

**Keywords:** educational psychology, higher education in prison, prison education, college-in-prison programs, incarcerated students, educational equity, critical paradigm

## Abstract

Research has well documented the far-reaching benefits of providing educational opportunities for individuals who are incarcerated, applicable to the students themselves and society. Given the many benefits, it is encouraging that access to U.S. Pell Grants for incarcerated students was restored in July 2023—the first time in nearly 30 years that need-based federal postsecondary financial aid was available to individuals in U.S. prisons. Although Pell Restoration enables an increasing number of colleges and universities to provide higher-education-in-prison (HEP) programs, this funding guarantees nothing about the quality and rigor of programming. In fact, relatively little is known about the nature, scope, and quality of HEP programs within the United States, and it is both timely and important to deeply examine these topics. The present study is a critical qualitative case study of three college-in-prison programs in the southern United States. To interrogate the nature and quality of the programs, this study explores the experiences and practices of program faculty and directors, drawing from research and scholarship in education and the behavioral sciences to examine two key areas: faculty training and the educational experiences made available to students. Multiple forms of data were collected, and two main findings emerged: (a) faculty training is piecemeal and limited, and (b) the educational experiences made available in the three programs are simultaneously empowering and disempowering. Using Ladson-Billings’s concept of the education debt (including its historical, moral, and economic underpinnings), this study highlights that the three college-in-prison programs—like many HEP programs across the United States—both contribute to and challenge the education debt.

## 1. Introduction

The United States, with over 1.8 million people held in state and federal prisons, has both the largest incarcerated population worldwide and one of the highest incarceration rates (Sawyer & Wagner, 2025). As is well known, the U.S. criminal legal system disproportionately impacts individuals and communities of color. For example, while White individuals make up 58% of the U.S. population, they constitute 36% of the incarcerated population. In contrast, Black individuals make up 13% of the U.S. population but 37% of those incarcerated (Sawyer & Wagner, 2025). Additionally, most incarcerated individuals come from educationally underserved communities.^1^ Before going to prison, less than half (38%) of people in state prisons completed high school, whereas almost 90% of adults in the U.S. have graduated from high school (Wang et al., 2022). This disproportionality in the data has remained consistent for decades (L. M. Davis et al., 2013; Miller, 2021).

Addressing mass incarceration in the United States—and all of its harms (Alexander, 2012; Gottschalk, 2016; Schenwar & Law, 2020)—is one of the most essential needs for ensuring just and equitable living conditions and opportunities. While we strongly support efforts to find alternatives to mass incarceration, we likewise highlight the importance and value of providing quality education opportunities to those who are currently incarcerated. Quality education increases employment opportunities, strengthens community within and outside of carceral spaces, promotes personal growth and development, can promote intergenerational benefits, and is a more effective and humane use of public resources than funding mass incarceration (Binda et al., 2020; L. M. Davis et al., 2013; Dewey et al., 2020; Milner et al., 2024; RAND Social and Economic Well-Being, 2025; Northwestern Prison Education Program, 2025).

Educational opportunities for incarcerated individuals have also allowed roughly 27% to obtain a General Educational Development (GED) credential (Couloute, 2018)—which is important in and of itself for educational attainment purposes, but also crucial because it makes one eligible for postsecondary education. Overall, the potential impacts of postsecondary educational opportunities for incarcerated students are compelling. As one student described:

Although vocational training and certification provides the incarcerated student with the requisite skills to make a living, the breadth of knowledge and accompanying consciousness that students may develop as a result of a liberal arts education provides that same student with the necessary utensils to make a life. As a student who has been incarcerated for over twenty years, I can personally speak to the transformative nature of a liberal arts education

(Castro et al., 2015, p. 18).

Based on having a high school diploma or equivalent credential, approximately two thirds of incarcerated individuals in the US are eligible to enroll in postsecondary coursework (Bacon et al., 2020). It is thus discouraging to find that programs are not reaching enough potential students. For instance, in a large-scale national evaluation, Taber et al. (2024) found that in 24 jurisdiction-wide systems of corrections, college-in-prison programs enrolled less than 5% of eligible and interested students in HEP programming, and another 16 jurisdictions only enrolled 5–9% of those who are eligible and interested. Moreover, the Program for the International Assessment of Adult Competencies (PIACC) Survey of Incarcerated Adults found that out of a nationally representative sample of 1351 incarcerated individuals between the ages of 16 and 74, 70% indicated an interest in participating in college coursework (Rampey et al., 2016)—a significant discrepancy from the percentage who actually obtain access to this educational opportunity. As Lockard and Rankins-Robertson (2011) argue, denying postsecondary education to incarcerated students violates the fundamental, internationally recognized principle of education as an intrinsic right. A diverse range of stakeholders (e.g., policymakers, education researchers and practitioners, civil rights activists, religious leaders, etc.) have likewise highlighted the moral, societal, and economic harms of mass incarceration, including denying incarcerated individuals opportunities for formal and quality education (e.g., Policy Research Associates, n.d.; Taber et al., 2024; Taylor et al., 2021).

### 1.1. College-in-Prison Programs and Policies in the United States

Inadequate access to college in U.S. prisons is a result of more than fifty years of tough-on-crime policies (Newell, 2013), which culminated in the 1994 Violent Crime Control and Law Enforcement Act. The Act eliminated state and federal prisoners’ access to Pell Grant funding, contributing to the near disappearance of prison-based college programs^2^ (Castro et al., 2015). That said, federal policy changes in more recent years have allowed for modest growth in prison-based higher education opportunities.

In 2015, the U.S. Department of Education (DOE) temporarily removed the ban on Pell funding eligibility for a small proportion of incarcerated individuals for the first time in two decades under the Second Chance Pell (SCP) pilot program. The program initially granted federal funding for 64 colleges in 28 states to enroll incarcerated students meeting specific eligibility requirements based on type of conviction and length of sentence (Castro et al., 2024). By the last year of the program in 2022, SCP had enabled more than 40,000 students to enroll in postsecondary education while incarcerated and receive 12,000 credentials (Elfman, 2024; Taber et al., 2024). In December 2020, Congress passed the FAFSA Simplification Act, monumentally lifting a 26-year ban on Pell grants for incarcerated students. Pell Grant restoration took effect on 1 July 2023, with more than 750,000 people in prison currently eligible to enroll in postsecondary programming (Taber et al., 2024).

Although Pell Restoration will enable an increasing number of colleges and universities to provide higher education in prison programs, additional funding guarantees nothing about the quality and rigor of programming. As Castro et al. (2024) note, some programs have used the Pell grant “as a slush-fund of sorts, allowing institutions to collect Pell dollars while providing substandard instruction and curriculum with little to no state or federal oversight” (p. 542). As such, it remains critical to attend to the specifics of what is being offered in prisons across the United States, as well as how and by whom such instruction is provided.

### 1.2. Overview of Existing Research Examining College-in-Prison Programs

For the most part, research on college-in-prison has narrowly focused on the relationship between postsecondary education and correctional goals, particularly reduced recidivism (e.g., Armstrong et al., 2012; L. M. Davis et al., 2013; R. H. Kim & Clark, 2013). While these concerns are vital from a correctional perspective, they simultaneously mask the need to ask critical questions about the nature and quality of the higher education afforded to incarcerated students. By reducing higher education in prison to its correctional function, prevailing discourses shift attention from incarcerated students’ best interests to disputes over what education those marked for ‘correction’ deserve (Castro, 2018; Castro & Gould, 2018; Evans, 2019). Overall, recidivism rates are a legitimate concern for society, but educators must concern themselves with questions about the nature, quality, and equitable conditions of education for all students—and a focus solely on recidivism is likely to undermine evaluations of, and improvements in, the quality of education for students who are incarcerated.

Despite the majority of existing research focusing mainly on how postsecondary education may or may not address correctional goals (e.g., reduced recidivism), there is a small but growing and important body of work in more recent years that is illuminating key characteristics of higher education in prison (HEP) programs across the country. For example, several landscape reports (Gaskill et al., 2023; Royer et al., 2020; Royer et al., 2021) and the National Directory of Higher Education in Prison Programs (2023) provide a descriptive overview of programs, such as the types of postsecondary institutions offering HEP programs, modes of instruction (face-to-face, virtual), credential pathways offered, and available student support services, among other key details. Additionally, some researchers have collaborated with currently incarcerated students to produce scholarship aimed at understanding and critiquing HEP programs in an era of mass incarceration (Castro et al., 2015); some have interviewed formerly incarcerated individuals who took college-in-prison programs to gain insights about the impacts or needs of HEP programs (Binda et al., 2020; Milner et al., 2024; Pelletier & Evans, 2019), while other papers have included self-reflections (McGuire, 2017) or more structured autoethnographic methods (Roberson & Alexander, 2022) from HEP program instructors to comment on their experiences as educators in prison contexts.

Similar to the present study, we also identified two reports where the research team interviewed college-in-prison educators or directors (Dewey et al., 2020; Royer et al., 2023) to better understand the context, practices, and/or challenges within some HEP programs located in the United States. Dewey et al. (2020) did not specify their research design, but their overall qualitative approach and subsequent report make a meaningful contribution by “capturing a snapshot of prison education” (p. 60) through conversations, observations, or shadowing of prison administrators and educators. They specifically sought to understand how, at a state-level, administrative approaches or modes of delivery for education and psychosocial programming reflect evidence-based practices. Although it was not their goal to specify details about their participants or data collection methods, they gathered important state-level information about HEP programming and provided several suggestions based on the information obtained. For instance, they highlighted potential benefits of (a) promoting peer-driven learning environments or strengthening collaborations with local universities and (b) providing HEP opportunities for those who are incarcerated regardless of age, sentence length, or conviction type.

Royer et al. (2023) focused their study on understanding the prevalence of disciplinary power within HEP programs and the extent to which it impacts students’ experiences in such programs. They conducted interviews with 19 individuals connected to prison education in some way, including program directors, instructors, family members of incarcerated students, and program alumni. Although the focus was not solely on college-in-prison educators and directors, their inclusion in the sample contributed to an overall finding in the study, which suggested that prison officers frequently obstruct students’ opportunities and success with HEP programs.

Collectively, although important research has emerged exploring HEP programming beyond its relationship with common variables such as rates of recidivism, researchers continue to argue that relatively little is known at this time about the nature, scope, and quality of existing programs (Binda et al., 2020; Castro et al., 2018; Milner et al., 2024). As Castro and Gould (2019) note:

higher education in prison programs have always existed with some level of secrecy, whether because of public opinion or stigma on campus, or other very real and emergent reasons. Without much of this descriptive information, we can actually say very little of the quality of programs

(p. 8).

Considering the gaps in existing research as well as the relatively recent reinstatement of Pell Grants for incarcerated students, it is both timely and important to deeply examine the nature of college-in-prison programs, as well as the extent to which they adhere to quality standards of higher education.

### 1.3. Conceptual Framework

Several sources of scholarship and theory within the behavioral and educational sciences inform this study, but for the purposes of this paper, we focus on the following areas. We begin by briefly describing how this research draws from the critical paradigm. We then present an overview of education debt (2006), also highlighting it as the overarching analytical framework for this study. Subsequently, we elaborate on other key components of the conceptual framework—including critical HEP scholarship on faculty training and prison pedagogies, as well as higher education research and scholarship on student engagement.

#### 1.3.1. The Critical Paradigm

The present research is guided by the critical paradigm, of which the central aim is to “critique, interrogate, and transform any system implicated in the oppression of humans” (Gonzales et al., 2018, p. 505). In examining how societies are organized and maintained—and to whose benefit—critical researchers foreground issues of power (Crotty, 1998; Freire, 1970/2000; Hooks, 1994; Hill Collins, 2008; Martínez-Alemán, 2015; Pasque & Carducci, 2015; Zald, 2002). In the spirit of critical research, we consider the material and ideological implications of program faculty and director practices on the nature and quality of college-in-prison programming in the southern part of the United States.

At the same time, we acknowledge that program staff practices cannot be disentangled from the broader carceral context itself, where prisons function as constraining institutions that not only shape the possibilities, limits, and meaning of higher education in prison but also limit incarcerated individuals’ agency, choice, and power in virtually every realm of their lives—including education. Indeed, an extensive body of work on prison sociology and criminology highlights prisons as complex systems of power where control extends far beyond physical confinement (e.g., Crewe, 2011, 2015; Goffman, 1961; Wooldredge, 2020). Goffman (1961) described prisons as “total institutions” that strip individuals of their autonomy. Crewe (2011, 2015) theorized “soft power,” the various psychological, relational, and procedural forms of control prevalent in carceral contexts—also describing prison life as a complex layering of power, uncertainty, and compliance. Even given the constraints and violence of the carceral context—and aligned with critical scholars and practitioners working in this area of education (e.g., Castro & Gould, 2019; Erzen et al., 2019)—we adopt the perspective that higher education in prison ought to and can be of high quality. We seek to elevate awareness about the postsecondary education afforded to underrepresented students in a nontraditional context that remains largely invisible in education discourse and policy on equity, inclusion, and quality. We also aim to identify program strengths as well as shortcomings that exemplify “better than nothing” education (Castro & Gould, 2018).

#### 1.3.2. Education Debt

Applying a critical race theory lens, Ladson-Billings (2006) reframes the so-called achievement gap in K-12 schools as an education debt to underscore the “persistent inequality that exists (and has always existed) in our nation’s schools” (p. 4). Ladson-Billings argues that a national focus on achievement gaps moves education toward short-term solutions that are unlikely to address systemic issues rooted in racism and oppression. Furthermore, she contends, the notion of an achievement gap places the blame of low educational attainment and underperforming schools on students and teachers rather than on the inequitable distribution of educational services and resources.

Education debt has four principal components: historical, economic, sociopolitical, and moral. Historical debt refers to the legacy of educational inequities in the K-12 system formed around race, class, and gender in the United States. Indeed, scholars have noted how these same inequities have plagued postsecondary education since its inception (Cabrera, 2014; Inwood & Martin, 2008; Wilder, 2013; Yosso, 2006; Yosso et al., 2004), and higher education in prison is no exception. Economic debt underscores the historical and contemporary funding disparities in education. Sociopolitical debt represents the degree to which families of color have been—and continue to be—excluded from the civic process, including from decision-making around their children’s access to quality schooling. Lastly, moral debt reflects the “disparity between what we know is right and what we actually do” (Ladson-Billings, 2006, p. 8). At the core of Ladson-Billings’s (2006) theorization of the education debt is a concern for the historical lack of access to quality education for individuals with low income, and especially for communities of color.

In the present study, we apply education debt to a higher education (in prison) context, and in doing so, we consider the implications of education debt for a novel group of students. Education debt undergirds the present study in two principal ways. First, given the disproportionate numbers of low-income and/or Black and Latinx individuals under incarceration, education debt underscores the urgent need to examine and address issues of quality with respect to education in prison, including postsecondary programming. Failure to do so means further perpetuating historical educational inequities and the implicit notion that only some students deserve a quality education. Second, education debt poignantly captures the educational histories and realities of many incarcerated individuals and helps to situate the general scarcity of diverse and high-quality higher education opportunities in prison—as well as mass incarceration more broadly—as extensions of a historical, racist, and classist system of educational marginalization and deprivation in the United States. Education debt also serves as a critical, analytical framework to engage with the broader implications of the present study. More specifically, the study’s findings will be interpreted in terms of how particular perspectives and practices within the college-in-prison programs under study exacerbate and/or challenge education debt.

#### 1.3.3. Higher Education in Prison

The present research is also guided by critical insights and critiques from the growing field of higher education in prison. Two main topic areas (briefly summarized in turn) allow us to highlight other central arguments undergirding this study: training of faculty who teach in prison, and prison pedagogies.

##### Training of Faculty Who Teach in Prison

In describing faculty training, Ginsburg (2019) stated that “the current practice in many programs is for adjunct professors to be hired to teach a given course in a prison, provide them a brief training on security matters, and then let them loose on incarcerated students” (p. 5). Critical prison education scholars and practitioners have rightly expressed concern for this lax approach to faculty training in many college-in-prison programs, arguing for more intentional and critical approaches to prepare faculty for the prison classroom (Ginsburg, 2019; Scott, 2013). Many have underscored the urgency for programs to help faculty understand the specific dynamics and inherent violence of prison contexts (Ginsburg, 2019; Scott, 2013; Wright, 2005). For example, incarcerated students must navigate a host of challenges and power dynamics in prison, not least of which include cramped living and study spaces, lack of control over noise and light, resource-deprivation, regularly surveilled movements and interactions, body counts, and cell inspections. As Wright (2005) highlights, without comprehensive pre-service training, faculty lack the cultural maps to understand their experience; they can only rely on stereotypical images of prisons in the media. Culture shock can ensue (Matthews, 2000; Wright, 2005), making the experience of teaching in prison unsustainable for some.

Yet thoughtful faculty training is not merely a matter of raising awareness about the realities of prison teaching; without it, faculty are “more likely to take on the values and imperatives of the carceral state” and become an added source of oppression for incarcerated students (Ginsburg, 2019, p. 1). Scott (2013) warns of the brand of positivism long prevalent in education where teachers predominantly concern themselves with curricular proficiencies while remaining ignorant about issues of power and privilege. As such, training must encourage and prepare faculty to be reflexive about their positionality, including how they occupy a place in the prison’s power structure (Ginsberg, 2019; Ginsburg, 2019; Scott, 2013). Furthermore, individuals working in the realm of college-in-prison must necessarily approach the work with humility—the job of a prison educator is neither to reform nor to save (Ginsberg, 2019; Ginsburg, 2019; Simpson, 2019).

##### Prison Pedagogies

Various prison educators and scholars have highlighted the prison classroom as a space holding transformative and liberatory potential (Castagnetto & Shanley, 2019; Erzen, 2016; N. B. D. Moore, 2015; Trounstine, 2008). For example, as an instructor of African history, N. B. D. Moore (2015) described his in-prison classes as an opportunity for some students to engage their African heritage and build community inside and outside of the classroom. Similarly, Castagnetto and Shanley (2019) explained that writing classes in prison can help to build community, create solidarity, and foster new life narratives through restoring. Multiple works co-authored by incarcerated and non-incarcerated scholars note similar ideas (Castro et al., 2015; Heider & Lehman, 2019; Roberson & Alexander, 2022). Castro et al. (2015) and Heider and Lehman (2019) underscore the potential for higher education in prison to foster self-development, critical thinking, and foment positive, societal change impacting both the incarcerated and non-incarcerated.

At the same time, multiple prison educators and scholars have problematized this notion of the prison classroom as a transformative and emancipatory site, particularly regarding instructors’ use of critical pedagogy (Castro & Brawn, 2017; Ginsberg, 2019; Hicks Peterson, 2019; Kilgore, 2011; Scott, 2013). The ultimate aim of critical pedagogy (Freire, 1970/2000) is to foster critical consciousness—what Freire terms “conscientization”—whereby learners gain a deeper understanding of the world through naming and questioning systems of power and oppression. Critical pedagogues strive to create learning environments wherein through inquiry and dialogue, students become empowered to respond to or act against internal and external oppression (Ellsworth, 1989). Yet in a totalizing and authoritarian institution like a prison, such a practice remains untenable (Castro & Brawn, 2017; Ginsberg, 2019; Kilgore, 2011; Scott, 2013). Ginsberg (2019) asserted that critical pedagogy seeks to do the impossible within a carceral context in attempting to disrupt and reframe prisoner identity and agency. Further, he warned that the “incarcerated student’s identity as prisoner’ is unintelligible to the non-incarcerated instructor, making critical pedagogy’s insistence on student transformation epistemologically baseless” (p. 61). In a dialogue between a professor and an incarcerated college student, Castro and Brawn (2017) similarly described critical pedagogy in the prison as a tragic endeavor, noting that it “may run the risk of reproducing the power structure they [non-incarcerated instructors] seek to expose by neglecting to consider incarcerated students’ unique positionalities—specifically, their inability to freely access information and to exist in the world as independent thinkers” (p. 103). In order to address these contradictions, the authors argue for an “emplaced critical praxis” (Castro & Brawn, 2017) that facilitates engagement in critical discourse and practice while accounting for the subjectivities of incarcerated students and acknowledging the prison classroom as an inescapable extension of a broader system of oppression.

##### Student Engagement

In addition to critical scholarship from the field of higher education in prison, an understanding of student engagement is important to examine the educational experiences made possible by the three college-in-prison programs under study. For the purposes of this research, we adopt Kuh’s (2001, 2003, 2009) definition of student engagement as the extent to which students participate in educational activities that are linked to positive learning and development outcomes and the degree to which institutions of higher education foster environments that enable students to engage in such activities. In using this definition, we echo Harper and Quaye’s (2009) critique that the latter component—institutional responsibility—is particularly critical in considering the educational experiences of underrepresented and marginalized students for whom engagement in college is often far less accessible than for the traditional-aged, white, middle class student. This concern is perhaps all the more pressing in prisons where students’ use of time and space as well as their mobility are controlled and restricted at every turn. In this study, we consider the student engagement benchmarks from the National Survey of Student Engagement (NSSE, 2023), which include (a) level of academic challenge, (b) learning with peers, (c) experiences with faculty, and (d) supportive campus environment.

### 1.4. Study Purpose and Research Questions

No past studies have explicitly evaluated college-in-prison program quality through the lenses of faculty training and the day-to-day classroom experience provided to incarcerated college students. This paper highlights a comprehensive, qualitative case study of three college-in-prison programs in the southern part of the United States. Our purpose was to explore the experiences and practices of program faculty and staff, as a means of interrogating the nature and quality of their programs. The study was guided by the following overarching question: What do the experiences of college-in-prison program faculty and staff reveal about the nature and quality of the higher education made available to college students who are incarcerated? The following sub-questions were used to further focus the study: (a) What kind of training do faculty receive to teach in prison; and (b) What kind of educational experiences are faculty and staff able to facilitate in prison? Faculty training as well as the provision or absence of particular educational experiences and opportunities in the three college-in-prison programs (e.g., access to resources and services on the traditional campus) provided important entry points for examining the nature and quality of the programs.

It is important to undertake a study of this nature not only to advance knowledge about the state of college programming in prisons, but also to identify and challenge potential instances of “better than nothing” education, wherein incarcerated students are systematically exposed to substandard educational experiences (Castro & Gould, 2018). This is particularly important given the historical and contemporary scarcity of quality, formal educational opportunities^3^ in prisons combined with the lack of college choice afforded to postsecondary students under confinement (Castro & Gould, 2019).

## 2. Method

### 2.1. Qualitative Case Study Design and Rationale

This study employed a qualitative case study research design. Qualitative inquiry is appropriate for exploring phenomena and populations that are little researched (Creswell, 2014; Creswell & Poth, 2018) and case study is well suited for exploratory research as it allows for in-depth understanding of social phenomena using multiple forms of data collection (Bartlett & Vavrus, 2017; Creswell, 2014). This study employed what Bartlett and Vavrus (2017) refer to as homologous horizontal comparison, wherein the entities under examination have a corresponding position or structure to one another (in this case, credit-bearing, prison-based higher education programs in a southern state of the United States). All study procedures were approved by the Institutional Review Board of the first author’s institution.

#### 2.1.1. Case Selection

To select the cases for this study, several criteria were used, including that the program must (a) be credit-bearing; (b) be provided by an accredited, nonprofit institution of higher education; (c) provide face-to-face instruction; (d) operate in one or more state prisons; (e) have been in operation for at least one year; and (f) only enroll incarcerated students. Of the seven total programs in the state that qualified, we narrowed selection down to three using a maximum variation sampling approach, wherein the cases represent a range of variation on a dimension of interest (Bartlett & Vavrus, 2017). Specifically, we selected college-in-prison programs representing a diversity of postsecondary institutional types offering college-level coursework in prisons in one southern U.S. state, not only to enable some degree of breadth but also to honor the different types of institutions working in this underserved area of higher education. In describing our three cases, all program names have been given pseudonyms to maintain program and participant confidentiality.

#### 2.1.2. Case 1: Lake Community College

The first case is a college-in-prison program offered by Lake Community College (LCC). LCC’s program is funded in part by the state Department of Corrections and state funding for community colleges. The program offers an Associate of Applied Science (A.A.S) degree in Heating, Ventilation, and Air Conditioning (HVAC) in one medium custody men’s prison. Coursework spans technical curricula and humanities. Students receive 30 h of instruction per week and complete five semesters of coursework. To participate, students must: (a) have a GED or high school diploma, (b) be in their final three to five years of incarceration, and (c) have been a resident in the state for at least one year so the program could pay in-state tuition. At the time of our study, the program offered two tracks: HVAC and Electrical Systems Technology. After completing the five semesters of coursework, students can get an A.A.S. in Air Conditioning, Heating, and Refrigeration Technology.

At the time of our study, there were 15–20 students enrolled in the LCC program. Students were male, between the ages of 18 and 85, with the majority in their 30 s. Across multiple semesters, the race and ethnicity breakdown of students has been approximately 50–55% Black, 40–45% White, and 5–10% Latinx.

#### 2.1.3. Case 2: River State University

The second case is a college-in-prison program offered by River State University (RSU), a public four-year, research-intensive institution. RSU’s program grants college credits but does not confer certificates or degrees. The program curriculum focuses largely on the Humanities and Social Sciences, spanning English, Literature, Writing, Sociology, Art History, History, and Cultural Studies. One quarter of the courses are writing courses to ensure that students strengthen written communication skills. All courses are taught face-to-face.

A typical semester in RSU’s college-in-prison program lasts 7.5 weeks, with classes scheduled to meet twice a week for approximately 2.5 h each class meeting. To participate, students must: (a) have a GED or high school diploma, (b) take the Wide Range Achievement Test (WRAT), and (c) have 10 years or less remaining on their sentence; if seats are available, someone with a longer sentence—including a life sentence—can participate. The faculty participants in this program teach in six different prisons, the majority of which are minimum custody; five of the six prisons are male facilities. Across the six prisons, students’ ages range from 18–65, with the majority in their 30s–50s. Student race and ethnicity across prisons is roughly 40–60% Black, 40–60% White, and 5–10% Latinx or Asian.

#### 2.1.4. Case 3: Mountainside University

The third case is a college-in-prison program offered by Mountainside University (MU), a four-year private university. The program offers an Associate of Science in Behavioral Science and is meant to help students pursue a Bachelor of Social Work and/or move into a range of helping professions. Coursework spans a wide range of areas, including English, Math, Religion, Social Work, Exercise Science, Psychology, Fine Arts, and Sociology. The program has traditional 15-week semesters, with classes meeting once a week for three hours each time. Classes are provided via face-to-face instruction.

The MU program is held in one male medium security prison. At the time of the study, 11 students were enrolled in the program, all between 25 to 50 years of age. The race and ethnicity breakdown of students was approximately 70% Black, 20% White, and 10% Latinx. To participate in the program, students must first undergo multiple screenings from the Department of Corrections (DOC) about behavioral issues, time remaining on sentence, and work release readiness. Once approved by the DOC, students must apply for admission to MU. MU requires that students (a) have a GED or high school diploma and/or submit a high school transcript and standardized test scores (if available), (b) complete an interview, and (c) have no less than seven or eight years remaining on their sentence.

### 2.2. Participant Selection and Recruitment

Both HEP faculty and program directors were recruited as participants in this study, all of whom worked within one southern state in the U.S. The following inclusion criteria were used to recruit faculty and program director participants: (a) taught in a credit-bearing higher education program in prison, (b) taught or managed for at least one semester in the program during the previous two years, and (c) was from an accredited two- or four-year institution of higher education. Across the three programs, 21 people participated in the study (18 faculty and three program directors).

#### Participants

Table 1 indicates the demographic characteristics of study participants by program. As the data indicate and is true of most college-in-prison administrators and faculty across the United States (Ginsburg, 2019), the majority of participants (85.7%) in this study self-identified their race/ethnicity as White. Additionally, just over half (57.1%) identified as a woman, ages of participants varied, and the median years working in higher education ranged from 7–15 years across the three programs—but in each program, the median years working in prison-based education was less.

Several steps were taken to protect the rights and confidentiality of all participants. For example, we obtained approval from the relevant university IRB, received consent from all participants, assigned pseudonyms to each participant and their respective institution, fully disclosed researcher background and intention to conduct research, and explained how participant confidentiality would be maintained.

### 2.3. Data Collection

Rigorous qualitative case study research integrates multiple forms of data collection to enable methodological triangulation and to strengthen the credibility of findings (Stake, 1995; Yin, 2018). In this study, we collected data through demographic and professional background questionnaires, semi-structured interviews, classroom description worksheets, as well as program and course documents. Demographic and professional background questionnaires elicited background information on participants to enable contextualization of interview responses and study findings. Semi-structured interviews obtained their in-depth descriptions and perspectives of the educational experiences afforded to students in their respective programs. Classroom description worksheets complemented participant narratives through staff descriptions of the learning environments made available to students. Document analysis supplied insight into institutional logics, policies, and program materials that shaped program design and delivery. Taken together, these methods provided a multifaceted understanding of the programs under study.

#### 2.3.1. Demographic and Professional Background Questionnaire

As a first step, each potential participant was asked to participate and directed to an informed consent form. Across all three programs, 100% of invited participants completed the consent form. Participants were then asked to complete a demographic and professional background questionnaire, which was distributed via a secure Qualtrics form.

#### 2.3.2. Semi-Structured Interviews

Interviews were the primary method of data collection in this study and were facilitated by the first author. One-on-one semi-structured interviews were conducted with each participant to capture key information and were guided by recommendations from Merriam and Tisdell (2016). Twenty interviews were conducted via Zoom and one by phone (at the participant’s request). All interviews lasted 60–90 min, were audio-recorded with the participant’s consent, and took place in a private setting. The interview protocol included questions about training and supervision (e.g., Can you talk about any training you received before starting to teach in prison? Can you tell me about any kind of supervision you received while teaching in prison?), as well as questions about specific classroom and pedagogical practices. For example, participants were asked “Do you do any peer-to-peer work in your course, and if so, what does that look like?”; “Can you talk about what kind of access students have to faculty both inside and outside of the prison classroom?”; and “Can you talk about any challenges you’ve experienced with teaching your course in prison?”

#### 2.3.3. Classroom Description Worksheets

As prison regulations did not allow us to observe the classrooms in each program, we asked each participant to complete a classroom description worksheet, in which they sketched their prison classroom(s) and shared their observations. In qualitative case study research, observation is most valuable when it includes both descriptive detail and reflective commentary (Creswell & Poth, 2018; Merriam & Tisdell, 2016; Stake, 1995). Descriptive observations included descriptions of the physical setting and environment while reflective observations included descriptions of how the physical setting and environment impacted teaching and learning. Although all participants were invited to complete the classroom description worksheet, 14 (67%) did so. Participant responses were compiled by program and used to develop composite descriptions of the classrooms in each program.

#### 2.3.4. Documents

We gathered and reviewed a total of 130 program- and course-related documents/files from study participants, the majority of which were course syllabi, assignment and project prompts, and lecture slides.

### 2.4. Data Analysis

Consistent with qualitative case study methodology (Bartlett & Vavrus, 2017; Creswell & Poth, 2018), we conducted “within-case” analysis to deeply examine the individual details and unique characteristics of each case, as well as “cross-case” analysis to compare those cases with each other. This approach allows for a better understanding of the phenomenon under investigation by capturing both context-specific nuances (e.g., exploring intricate relationships between different elements within a case to better understand its specific context) and identifying similarities, differences, and/or patterns that may appear across the entire sample.

Interviews were analyzed using thematic analysis. All interviews were first transcribed and subsequently uploaded into QDA Miner Lite, a free Computer Assisted Qualitative Data Analysis (CAQDAS) program. A broad scan of the interview data corresponding to each program was conducted, taking initial notes in a hard-copy research journal about general ideas, overall tone, and depth of information.

Two cycles of coding were utilized and followed recommendations by Saldaña (2016). The first cycle coding included two rounds of open-ended and eclectic coding. Text was analyzed by paragraph and research question, using three types of codes: (a) in vivo codes, which are verbatim words or short phrases from interviews; (b) descriptive codes (e.g., descriptions of training or teaching); and (c) a priori codes generated from the conceptual framework and relevant literature (e.g., learning with peers). These three types of codes allowed the authors to address the research questions, take note of key descriptive information, incorporate the voices of study participants, and stay close to the conceptual framework.

The documents received from participants were also coded using QDA Miner Lite, employing the “variables” tool to create categories of documents corresponding to each participant (e.g., syllabus, assignment, campus magazine article). Before coding documents, their authenticity was assessed (McCulloch, 2004) using suggested questions from Guba and Lincoln (1981), including who the author is (e.g., the faculty member teaching a class), for whom the document is intended (e.g., an assignment intended for students), the sources of information provided, and possible sources of bias.

Second-cycle coding was then conducted to continue focusing the coding scheme. Pattern coding (Saldaña, 2016) was utilized to group individual codes (e.g., library access, study hall, tutoring) into larger categories (e.g., resources and services for students) and subsequently into broad themes corresponding to each research question (e.g., resources and services for students are a work in progress). This process allowed for in-depth, within-case analyses and the development of preliminary themes by program. Across-case analysis was subsequently conducted to establish final themes across programs and by research question. As a final step, the findings were read through the lens of education debt. More specifically, narratives were coded within themes using the four previously discussed components of Ladson-Billings’s (2006) concept of education debt: historical, economic, sociopolitical and moral debt.

Except for the demographic questionnaire (which was used to gather background information about all participants), data triangulation strategies were used with all other sources of data. For example, data from interviews, documents, and classroom description worksheets were cross-referenced to identify areas of possible convergence and divergence. With the exception of minor details that were inconsequential to the study’s findings, the analysis revealed strong consistency across data sources. For instance, in the RSU program, faculty accounts of the information and guidance they received (or did not receive) before starting to teach in prison were corroborated by a program orientation document shared by a faculty participant. By systematically comparing and integrating multiple data sources, the study minimized reliance on any single perspective, enhancing the credibility and trustworthiness of its findings (Creswell & Poth, 2018; Lincoln & Guba, 1985).

### 2.5. Trustworthiness of Data

To address aspects of trustworthiness and credibility when conducting a qualitative study (see Bartlett & Vavrus, 2017; Merriam & Tisdell, 2016), we employed multiple strategies. First, we thoroughly disclosed the data collection and analysis strategies employed throughout the study. Second, consistent with standard recommendations (e.g., Maxwell, 2013; Merriam & Tisdell, 2016) we shared the main findings—the final cross-case themes as well as within-case findings—with participants in each program to ensure their perspectives and descriptions were captured accurately in our final results. Third, we triangulated the findings using multiple methods of data collection (questionnaires, interviews, classroom description worksheets, and documents) and multiple sources of data (e.g., cross-checking interview data collected from different people with divergent perspectives). Fourth, we provided rich descriptions of the settings and participants to the extent possible (given confidentiality issues), allowing readers to appropriately contextualize study findings and determine the level of transferability to other places and programs (Lincoln & Guba, 1985; Saldaña, 2016). Fifth, the sources and purpose of the documents collected for the study were reported in this paper to provide readers with proper context and background. Last, as recommended for this study design (e.g., Bartlett & Vavrus, 2017) all data were comprehensively analyzed. For example, we analyzed data until reaching saturation and we sought (and noted during the data analysis process) both confirming and disconfirming information.

## 3. Results

Data analysis from this qualitative case study revealed two primary themes. Each is summarized in turn and serves to address our research questions about faculty training and the educational experiences facilitated within the college-in-prison programs under study. For purposes of brevity, we provide only a few illustrative examples within the specified themes; however, interested readers can contact the corresponding author to request additional details.

### 3.1. Theme 1: Faculty Training Is Piecemeal and Limited

Faculty training is an important aspect of higher education regardless of context, including but not limited to college-in-prison programs (Ginsberg, 2019). This study revealed the piecemeal and limited nature of faculty training across programs. Specifically, the only formal training that faculty received was from the respective prisons, and it focused solely on security-related policies and safety. Most faculty shared that in addition to the policies and safety training, they received general and logistical guidance from program directors. Faculty in the RSU program highlighted informal sharing of resources with faculty who had previously taught in their programs. Across programs, the majority of faculty and program directors expressed the desire and need for more training. In the following sub-sections, we elaborate on each of these areas.

#### 3.1.1. Formal Training Focused on Security Policies and Safety

Across all programs in this study, all faculty shared that the only formal training they received to teach in prison was provided by the prisons and focused primarily on security-related policies and safety. In particular, faculty received an orientation and training on the Prison Rape Elimination Act (PREA), a law designed to address and prevent sexual violence in prisons as well as to institute zero-tolerance policies for sexual assault (PREA, 2003). According to Lauren at LCC, “PREA is all about creating boundaries between instructors and students, warning signs, things to watch out for.” Though PREA training is mandated by law, in a few rare instances, faculty in each of the programs did not receive the training for various reasons, including having received the training in a prior year. Although prisons in the state are required to provide a refresher course on PREA every two years, one faculty member reported that it had been at least five years since her first and only training.

A handful of faculty across programs shared the ways in which their prison orientation and training emphasized discipline, control, and the potential threat of danger with respect to incarcerated students. For example, Mindy at LCC explained that she was told to “be stern and firm with them [students] from the get-go, lay out the rules…” Alexandra at RSU recalled, “They kept saying to me, they [students] are like middle-schoolers…you have to keep them busy, keep them active. Don’t let there be any downtime. Something bad’s going to happen.” With regard to concerns about safety, Vanessa at MU described:

I think what’s interesting about going in there is that I never thought about safety concerns until sort of being oriented from the corrections side… But having said all that, in the classroom I never ever felt remotely insecure with the students… I simply can’t conceive of these guys committing violence against me.

Many faculty across programs, including Mindy and Alexandra, echoed Vanessa’s sentiment that their experience working with students in prison rarely, if ever, resembled the ways in which correctional staff depicted classroom safety concerns. Faculty across programs uniformly described the notable absence of required training from the program side, likening their experience to being “thrown to the wolves” or a “baptism by fire.”

#### 3.1.2. Informal Guidance

Although there was no formal training from the various universities, several faculty at MU and RSU described meeting with their respective program directors to discuss basic logistics and policies before entering the prison classroom. For instance, Helen at RSU described:

He [the program director] briefs us on kind of what’s expected, the level of instruction that we’re expected to give, but also the lack of resources that we’ll have, who the population is you know, the facilities that we’re going to, we’re not going to the maximum-security facility or anything like that. He talks about getting into the prison because, for most of us, we’ve probably never even been inside of one as a visitor.

Indeed, a “Guide for Correctional Education Instructors” provided to each faculty at RSU (submitted as a document by one participant) shows that pre-service information focuses on the basics of teaching in prison, including the various required steps (paperwork that needs to be completed, class roster, entering grades, doing course evaluations); what to do about school supplies, travel, food for class; and classroom discipline and safety, particularly the need to report any type of harassment to the Department of Corrections. The Guide comes with an Academic Information sheet to be distributed to each student, with information about post-release education (phone numbers, websites, advising, policies, etc.).

The program director at MU similarly commented that in initial conversations with faculty, he focuses on safety, classroom facilities, services for students, and—like the prison—the importance of keeping appropriate boundaries with students. He noted:

I don’t want them to be best friends. I don’t want them [faculty] to be taken advantage of. I don’t want them to take advantage of, for example, the offenders also. So it’s that dual situation…You want to keep it professional…You want to keep boundaries, probably tighter boundaries here than you would in a regular classroom.

The program director at LCC described providing some informal guidance about the community college (some program faculty only teach in the prison) and the use of the learning management system in the prison.

Beyond speaking with the program director, faculty at RSU talked about ad-hoc sharing of materials and resources as informal preparation for teaching in prison. Anna shared, “We met a couple of times as instructors, groups of four or five, and we would talk about the syllabus or we would talk about others’ materials.” Cameron described similar instances of resource-sharing, but also importantly highlighted that “faculty meetings are not trainings, it’s like skill share or here’s a new idea or this is a new thing we’ve got to worry about.”

#### 3.1.3. Need for Further Faculty Training

The majority of faculty and program directors across all three programs expressed the need and desire to do more with faculty training. Although study participants in each of the programs had myriad suggestions for training, across the three, various people highlighted the need for more preparation on logistics and the basics (e.g., entering and exiting the prison, rules on what can and cannot be brought in, dress code, rules for the classroom, prison schedules and procedures that may impact teaching, etc.). Multiple RSU and MU faculty and staff also emphasized the need for training on pedagogical issues, including teaching adult and non-traditional learners, teaching without technology, and accommodating students’ specific needs. Though in the minority, a few faculty felt that more training was not necessary; some professors in the RSU program described their experience teaching at the college level—including in community college where students had a diverse range of skills and backgrounds—as sufficient preparation for teaching in prison. Tom at MU noted that in addition to having ample years of experience teaching university students, he had taught in another prison in the state.

### 3.2. Theme 2: The Educational Experiences Made Available to Students Are Simultaneously Empowering and Disempowering

Education scholars and researchers have long theorized the concept of student engagement in higher education, highlighting particular factors that contribute to enhanced learning and development in college (Astin, 1993; Tyler, 1949; Pace, 1980, 1984). In this study, we drew in part on engagement benchmarks from the National Survey of Student Engagement (NSSE, 2023) to examine the educational experiences afforded to students in the three programs under study, including (a) level of academic challenge, (b) learning with peers, (c) experiences with faculty, and (d) supportive campus environment (particularly resources and services for students). Drawing upon insights from scholarship on higher education in prison, we also considered components of the educational experience outside of a student engagement lens, including community-building and opportunities for students to engage with their lived experiences and subjectivities.

Study results highlighted that the three programs in this study offer educational experiences that are both empowering and disempowering. Aligned with experiences being empowering, faculty strive for the coursework to be academically challenging and consistent with coursework outside of a prison context, in-class peer learning is valued, and classes provide unique opportunities for community-building and for students to engage with their lived experiences and subjectivities. However, at the same time, (a) meaningful out-of-class peer learning is only possible in the MU program; (b) interactions with faculty are mostly limited to in-class time; and (c) students have no access to the Internet, no direct access to university libraries, and have varied access to computers. The MU program offers several resources and services that the other programs do not. In the following sub-sections, we elaborate on these findings.

#### 3.2.1. Academic Challenge

The majority of faculty across programs described their in-prison courses as being generally comparable to what they offer on their non-carceral campus in terms of rigor, desired learning outcomes, and holding high expectations of students. Within NSSE’s (2023) student engagement framework, academic challenge encompasses various aspects of engagement, including two areas that were consistently highlighted through participant interviews, course syllabi, and assignment and paper prompts: (a) higher-order learning and (b) reflective and integrative learning. Briefly, higher-order learning entails in-depth synthesis and analysis of ideas; applying facts and theories to practical issues; and evaluating information sources. Faculty across all programs described numerous examples of higher order learning. For example, Greg’s literature courses in the RSU program center on “coherently and persuasively analyzing works of literature” and interpreting course readings with peers. Helen and Gwen’s art history courses focus on critical thinking and visual analysis. Helen noted:

They’re learning to critically think of images and think about the situations in which images are produced; who controls the production of images, how images can be manipulated, and how that affects our day-to-day-lives. And I think because we are such a visual society that that’s actually a more valuable skill than people tend to think of it as.

Vanessa, an English professor in the MU program, discussed facilitating critical and active reading through reading journals where students reflect on what they have read, and consider if and how it challenges their thinking or gives them a new perspective.

Faculty across programs also highlighted numerous examples of reflective and integrative learning, which encompass activities such as examining one’s beliefs and assumptions, considering diverse and alternative perspectives on critical issues, and combining ideas from different courses. For instance, Emma, a professor of Social Work in the MU program described:

I expect them to come out of it [the course] with a real critical look at how our society functions in a way that oppresses some and not others, and leads to the exclusion of many. And I do expect them to be able to look at it from both sides. You know, what beliefs do the oppressors have…Are they all just monsters?

David, the program director at RSU and a professor of cultural studies, shared that in his course students discuss issues of identity, subjectivity, race, and gender; engage with critical theory; and consider popular culture materials through these critical texts.

#### 3.2.2. Learning with Peers

Peer learning and collaboration is an important aspect of learning and development in college (Braxton et al., 2004; Lane, 2018). Across the three programs, the majority of faculty discussed regularly incorporating in-class peer work, mostly in the form of pair and group work as well as group discussion. Several faculty noted that because students in their programs tend to be older and have greater life experience than the traditional-aged college student, class discussions tend to be more involved and organic. Casey at RSU highlighted, “They [the students] know themselves and they are more open to participating and speaking and have more experiences to draw on. So the older thing I think is great.” NSSE emphasizes “discussions with diverse others”—in terms of race, ethnicity, class, religion, political views, among other markers of identity—as an important component of an engaging postsecondary experience. Various faculty in the three programs described how their coursework facilitates engagement with diverse perspectives. Matthew, a Professor of Religion at MU, described how his Introduction to Christianity class exposes students to religions outside of their own, noting that:

All of the students in the prison had a faith tradition and even those that are practicing…a unique African approach to the Muslim faith that is predominant in prisons…most of the students that are gravitating toward that even come out of a Christian tradition and most of them Protestant…it made for interesting conversation.

As a stark illustration of how a prison-based college classroom might facilitate meaningful interaction among people who may otherwise never engage with one another, Emma, a social work professor in the MU program, described having a student with a swastika tattoo on his forehead, noting “In any kind of group work, the former white supremacist has had to work with African American students.” She highlighted that although some prison officials expressed concern about in-class discussions about race due to the presence of racially segregated factions within the prison:

any time we talked about it [race], they all did pretty much agree with the facts presented to them. There wasn’t any, what about white men? What about our rights, kind of thing that I expected…There was nothing like that.

In general, Emma described in-depth and fruitful group discussions about systemic oppression, the impact of various intersectional identities on life experiences and opportunities, among other areas.

Although most faculty across programs described regularly incorporating peer work into their classes, a few RSU faculty described doing less or no peer work and provided different reasons for this. Helen explained:

I didn’t do too much group work in the prison setting just because I wasn’t sure how their dynamics are. I didn’t know who knew each other and who didn’t. I was not sure what the dynamics would be.

Cameron highlighted that peer work in class can be challenging because students often have little time for socializing outside of class:

I actually try to limit peer-to-peer work in the prison setting…I found that any kind of group work devolves extremely quickly into chatting because they have such curbed social lives that like any opportunity to talk, they’re going to try to just shoot the shit essentially.

Cameron added that the hypermasculine environment of prison can also make students uncomfortable with peer learning that requires them to share their work:

Nobody wants to share work with one another…I think it’s different in the women’s prison than it is in the men’s prison because of the way that people perform gender. There’s like razzing that goes on between the guys. A lot of times it will devolve into, I don’t care or they pretend not to care about the work as much as in the women’s prison because it’s not cool to really care about your personal narrative. It doesn’t give you any sort of capital in the social environment.

#### 3.2.3. Experiences with Faculty

Sustained and regular student-faculty interaction is an important aspect of a college experience (Cole, 2011; Y. K. Kim & Sax, 2014; Mayhew et al., 2016). Based on their accounts, it is clear that faculty in all three programs care deeply about—and are highly dedicated to—supporting students however and whenever they can, often times going above and beyond what is required of them.

Yet, unlike on a traditional college campus, access to faculty outside of class is not guaranteed. The majority of faculty across all programs described that they did not have the ability to interact with and support students outside of class. At best, there were brief moments outside of the class session that students could ask additional questions, including during class breaks, for a few moments after class, or walking out of the class together. Multiple faculty across programs shared different logistical barriers to helping students even at these times. Vanessa and Emma at MU noted that the remote location of the prison necessitated that they leave the prison relatively quickly after class in order to be able to make the next commitment. Some faculty at LCC and RSU added that because of prison schedules and/or correctional staff’s desire to leave immediately after class, they were unable to stay back and provide additional student support.

Several faculty described that they used in-class time to partially compensate for the lack of faculty access outside of class. For example, Casey in the RSU program explained:

Because two and a half hours is quite a long time for class…there’s often a rotating conference time. So people will just come up and talk with me one-by-one during class time while someone else is working on whatever. So I try to do that fairly often so that I can talk to everyone individually, and I do a lot of going around to everyone’s desk.

Anna employed a similar method to provide more individualized support:

The general format would be for the first hour we would talk about content and about what needed to be accomplished or what have you. And then the rest of it would essentially be an office hour where I would go around to each student and check on their work.

At the same time, various faculty across all programs noted that at times, prison schedules and procedures also constrained time with faculty during class. Matthew at MU provided an example:

I realized that my class wasn’t going to start at 8:00. I think on a good day, they [the prison] didn’t release them [students] until 8:00 to come to class. Then I was told after I got there that I needed to be finished by 10:15 because at 10:15 they have a count. And it shuts the place down and you don’t move…it could take up to 45 min before they open the campus up for you to be able to leave. So they were encouraging us to be out of there by 10:15. I thought I was going to have two hours and 45 min every week to deliver content. And now it’s cut down. So I was changing things quickly.

Other faculty also identified body counts, students’ in-prison work assignments, laundry days, and meal schedules, among other prison-related factors, as disruptions to in-class time.

#### 3.2.4. Supportive Campus Environment

Supportive campus environments have resources and services that help enhance student success—including academic libraries, academic advising, tutoring, and career counseling, among others (NSSE, 2023; Soria et al., 2017). In prison contexts, where education of any form is not the primary mission, resources and services for students remain a challenge. Participants highlighted this across two main areas: students’ access to a library and to computers and Internet. Additionally, the MU program offered some unique resources worth summarizing.

##### Library

One of the biggest concerns expressed by 100% of faculty and program directors was students’ lack of direct access to the electronic, campus library. Students in the RSU and LCC programs are only able to get resources from the library via faculty; faculty described the time-consuming process of compiling and bringing resources into the prisons for student use. At MU, students and faculty had support from campus librarians; librarians developed two types of request forms to enable students to ask for a particular book or journal article, or for requesting resources for specific research topics. Campus librarians then search for resources and either a librarian or the program director delivers the materials to the prison. According to the library request forms, the turnaround time for resources is one week. Not surprisingly, Vanessa and Emma at MU described the stress that this timeline often caused for students. They noted that in part, the process was slow because faculty were required to sign off on research requests on the one day a week that they saw students. In addition to these challenges, students’ ability to select different research topics and access a wide range of materials inevitably rely on the amount of time faculty or librarians are able to devote to finding resources. Many faculty described the significant amount of time they invested in doing this work.

##### Computers and Internet

All participants discussed students’ access to computers and Internet. In the MU and LCC programs, students are provided with computers. In the case of MU, students are provided with laptops to be used strictly in the classroom. At LCC, the classrooms are essentially computer labs where each student has a desktop computer. Still, Lauren at LCC explained that students cannot save their work because “within four weeks of a class ending, the computers are wiped and they [students] are not allowed to have flash drives. She commented, “I just thought, that’s not even humane. For me, if I did all that work, I would want copies of it.” As a result, Lauren tried to provide students with copies of their work whenever possible, noting that some students wanted it not only for themselves, but to send to their families.

Unlike in the MU and LCC Programs, students in the RSU program do not have access to computers. As a result, they have to handwrite everything, including papers that could be five to eight pages in length or more. Casey at RSU explained, “It’s frustrating for students and for me and they’re spending hours writing two copies of something.” Alexandra added:

It [handwriting papers] takes longer, it’s physical, you’re physically hurt afterwards. So it can be tough when they [students] have already done say, one or two drafts of their paper, they don’t want to write it again. And they don’t want to turn in anything where it’s like, this paragraph goes up here. Writing is this completely kind of iterative process where usually you write an ugly draft and then you revise. And you can reorganize.

Casey noted that the RSU program considered providing computers but ultimately determined that there were too many barriers to warrant the time investment in obtaining them.

Furthermore, none of the students in the three programs have access to the open Internet. At most, students at LCC have the ability to log into a learning management system. Generally speaking, across all programs, faculty discussed playing multiple roles in an attempt to compensate for the lack of—or limited—resources and services, including academic advisor, career counselor, and librarian. However, and understandably, various faculty noted that they often lacked the knowledge to provide sufficient assistance in these areas.

##### Unique Resources and Services in MU Program

Several unique resources and services were available to MU students that were not available to LCC and RSU students. For example, MU students have study hall five days a week for two to two and a half hours each day. Dedicated, quiet spaces for study are rare in most prisons (Simpkins, 2015; Sanford & Foster, 2006). Elise at RSU stated, “They [students] would just be like, look, you don’t understand how hard it is to work here, it’s so hard to find a good environment where you can sit down and read uninterrupted.” MU program faculty and the program director noted that as an added benefit, the study hall offers one of the few spaces where students and their activities are not under constant surveillance from correctional officers. In addition to study hall, the MU program director described establishing tutoring services in prison via the Student Support center on campus. Tutors are undergraduate students who have successfully completed a given course in which students in prison seek additional support. MU faculty and the program director also commented that the program has a general teaching assistant (TA) for all courses; the TA is an incarcerated individual with some higher education experience that can assist faculty with administrative tasks and provide homework assistance to students during study hall. Emma, a professor of social work, also noted that in her role, she is able in some respects to serve as both educator and social worker (informally) with students. She described supporting students through connecting them to resources and providing a listening ear:

As a social worker, I’m also working to really build these men up and…using the college class experience as a respite or safe place…Whereas in a regular college classroom there are a lot of things where I would normally just say, thank you for bringing that to my attention. How about we talk to somebody at the Counseling Center, whereas in the prison, they don’t have anybody else. So for example, there was one man whose wife…was extremely sick…And so he did ask me to approve research articles on this blood cancer.

Students cannot access library resources without approval from instructors, making Emma’s willingness to approve non-course related materials particularly important. Finally, one of the most critical supports and resources for MU students is Ed, the program director, who is on-site at the prison five days a week and meets regularly with students to answer questions, offer academic support (e.g., devise study strategies, etc.), and address any academic issues that may arise.

##### Beyond Student Engagement

As Harper and Quaye (2009) note, it is critical to consider, and move beyond, a traditional student engagement lens when examining the postsecondary educational experiences afforded to marginalized students. We describe two areas that faculty and staff in all three institutions highlighted about their college-in-prison programs: (a) community building and (b) opportunities for students to engage with their lived experiences and subjectivities.

Community building

Various scholars of higher education in prison have noted the critical role that college-in-prison programs can play in building community within prisons (Castagnetto & Shanley, 2019; Heider & Lehman, 2019; N. B. D. Moore, 2015). Across institutions, faculty indeed cited this aspect of their programs. Chris at RSU indicated that coursework provides the sense that “we’re in this together inside the classroom.” Several faculty across programs emphasized camaraderie as an important element of their classroom. Alexandra at RSU noted that in her course evaluations, multiple students described the “communal experience” and the “supportive and uplifting environment” as some of the best aspects of the class.

The RSU program director commented that community building in the prison-based college classroom is particularly meaningful: “Community is very difficult to achieve in prison. And then a classroom is a community…the contrast between that and what’s possible generally in prison, is pretty stark.” Casey in the RSU program added, “It really creates a little community and in the class they have something that isn’t controlled in the way the rest of prison is.”

Greg at RSU described community-building in a broader, global and historical sense. In reading Charlotte Perkins Gilman’s short story, the Yellow Wallpaper—in which the central character’s physician husband, believing she suffers from depression and hysteria, confines her to bed rest and forbids her from writing or working—a student commented that having been in solitary confinement, he could intimately relate to the experience of the protagonist. Greg commented that in engaging with themes of oppression in literary works, students often come to see:

that it’s not just them…there’s a whole history of this and other folks who have dealt with this. I think it creates a bit of solidarity, even with folks who aren’t alive anymore, but realizing that they’re part of the long struggle.

Faculty and staff across programs underscored aspects of community, camaraderie, and support that they observed—and participated in creating—in their classrooms.

Opportunities for students to engage with their lived experiences and subjectivities

In addition to community-building, faculty across programs emphasized providing opportunities for students to engage with their lived experiences. Casey in the RSU program noted that in her literature courses, they analyze systems of power and how they affect people’s lives. She noted:

We talked about racism, prison, policing, sexism, things like that. I want students to be able to understand and grasp the systems; most people can intuitively because of lived experiences. So it becomes more of a deeper discussion about nuances.

Casey also highlighted that she often prioritizes teaching literature written by currently or formerly incarcerated people while simultaneously noting that she also leaves space for people to explore beyond these boundaries, importantly highlighting that “it’s not just about, we’re in prison and let’s talk about prison. I try to balance that so that we have opportunities to think beyond the walls that we’re in.” Chris, a sociology professor in the RSU program, described a lesson, which focused on the impact of geography and residential segregation on people’s lives. He described:

We map out the racial composition of the city [where they grew up] in terms of zip codes and then I have them pinpoint …where they grew up and then we talk about how that shapes their lives. And, like in my on-campus class, people start to make the connections. They’re like, my individual-level experience is not just because of my work ethic or my psychological characteristics. It’s directly connected to the broader social forces.

Emma at MU described one of the several assignments in which she asked students to think like a social worker and consider issues that matter for local communities, including their own. She noted:

I would give them a neighborhood or a community situation…And they would have to talk about…if you were the social worker and you only had so many dollars to bring in some community programming, what would you bring in? They would talk about whether or not it would be to establish a food pantry or would they want to have a community meal. If it was an elderly housing complex something like a community meal might be a better idea…so they have to talk about how they would address food insecurity in this scenario.

In the LCC program, Lauren shared that her final project is a proposal for social change. She described one student’s proposal in which he explored the topic of securing higher compensation for state-appointed defense attorneys so that low-income populations could access fairer representation in the criminal legal system. In the RSU and MU programs, various faculty talked about how their courses focus on issues of race, racism, gender, subjectivity, and the history of people of color, which given the racial demographics of the prison classes—where 50–90% of students are students of color—is important and provide opportunities to think critically about subjectivity, history, and oppression, among other areas.

## 4. Discussion

*I refuse to create in myself, or promote in others, the idea that learning in prison sets me free without acknowledging the extent to which being educated in prison also helps me understand the extent to which I am unfree. *

*(James Davis III, Caught Somewhere Between…)*

The present case study serves as an empirical contribution to the relatively small knowledge base on postsecondary education in prison. The research, drawing from intersections of educational and behavioral sciences, is in part a response to calls in related disciplines to center questions of nature, scope, equity, and quality in examinations of college-in-prison programs (Castro et al., 2018; Castro & Gould, 2019; Erzen et al., 2019).

Overall, this study demonstrates that college-in-prison programs operate within a dual reality: while they reproduce and even exacerbate entrenched educational inequities, they also create rare spaces of intellectual growth, human connection, and community. This paradox underscores the urgent need to interrogate how program structures, policies, and practices simultaneously constrain and cultivate possibility. In the following sections, we use the framework of education debt (Ladson-Billings, 2006) to examine how faculty preparation, classroom experiences, and structural limitations of higher education in prison reflect and reproduce historical, economic, and moral debts—even as moments of humanizing education emerge within these settings. We also engage with critical scholarship specific to education in prison, and research on student engagement in higher education.

Although the present study facilitates a critical examination of three college-in-prison programs, program critiques are not intended to summarize individual programs as good or bad. Such an analysis would not only be a gross oversimplification but would fail to acknowledge how these programs collaborate with, and rely on support from, their sponsoring institutions of higher education, the respective prisons, the state DOC, and state and/or private funding. In discussing our findings, our objective is to provide in-depth understanding of these programs, highlight strengths, and call attention to flawed, harmful, or absent *practices* in need of addressing.

### 4.1. Faculty Training and Historical Debt

The report, “Equity and Excellence in Practice: A Guide for Higher Education in Prison,” highlighted the need for college-in-prison programs to “dedicate significant time and attention” (Erzen et al., 2019, p. 5) to the recruitment, training, and supervision of all program faculty and staff. Critical prison education scholars and practitioners have rightly expressed concern about the limited nature of faculty training in many college-in-prison programs, where at best, instructors receive a brief training on security matters before starting to teach in prison (E. Corbett, personal communication, 5 January 2021; Ginsburg, 2019). Consistent with these observations, faculty at RSU, LCC, and MU reported that the only formal training they received was from the respective prisons on facility policies and safety and that beyond that, faculty only received general and logistical guidance from program directors or select resources from faculty who had previously taught in their programs. This norm is all the more troubling when one considers the racial demographics of most prison classrooms across the United States, including those in this study—the majority of faculty participants identify as White, while generally speaking, their students across multiple prisons are at minimum 50% individuals of color and often times more, with Black students overrepresented among students of color. As Dr. Erin Corbett, long-time prison educator, advocate, and administrator; researcher of prison higher education; and founder and CEO of Second Chance Educational Alliance, asked in a recent conversation, “How do White faculty prepare themselves to go into spaces that are largely populated with Black and Brown bodies?” (E. Corbett, personal communication, 5 January 2021).

Lack of comprehensive and thoughtful faculty training in the college-in-prison context is reminiscent of—and extends upon—a long history of K-12 teacher preparation programs that have been largely unsuccessful in training a predominantly white teaching force to work effectively with increasingly racially diverse student populations (Goldenburg, 2014; Love, 2019). We thus frame the lack of formal faculty training on matters beyond safety and security-related policies in the programs in this study—and in many college-in-prison programs across the country—as an exacerbation of historical debt. Ladson-Billings (2006) described historical debt as the legacy of educational inequities in the K-12 system formed around race, class, and gender in the United States. Ample scholarship highlights that K-12 teacher preparation programs often fail to build the critical and intercultural consciousness of white teachers (e.g., Bloom et al., 2015; Milner, 2010; Milner & Laughter, 2015). Programs often do not sufficiently address issues of identity, race, class, culture, and systemic oppression, among other areas of importance. Many K-12 teachers leave preparation programs never having—or only superficially having—examined their beliefs, practices, or positionalities. As such, they may project deficit-based thinking and other harmful biases onto students of color (e.g., Bloom et al., 2015; Howard, 2010; Hyland, 2005). Likewise, the college-in-prison programs in this study as well as many others across the country have not facilitated opportunities for faculty to engage critically with relevant issues (e.g., mass incarceration, the history and role of education within prisons) or to reflect on “how race, class, gender, ability, sexuality, and other identity or status markers might impact their interest in, or approach to, teaching in prison” (Erzen et al., 2019). Andra Slater (Castro et al., 2015), who completed college coursework while incarcerated commented:

Upon entering prisons, white prison educators come into contact with people and communities of color who have radically different backgrounds than their own. I wonder if they have ever grappled with their own deeply held ideas and assumptions about those of us who are incarcerated

(p. 25).

Ginsburg (2019) argues that in the prison classroom, as in any classroom, “an honest appraisal of one’s assumptions, prejudices, and biases will contribute to better classroom experiences, and a more trusted and sustainable program” (p. 7). The absence of thoughtful faculty training in HEP programs can result in prison educators’ own brand of deficit thinking or what Slater (Castro et al., 2015) described as the “wow factor” (p. 25), wherein prison educators are wowed because students’ intellectual abilities surpass what they expected to find inside prison. Without comprehensive training, some faculty may also lack awareness of the dynamics and the inherent brutality of prisons and how that environment impacts students’ lives and learning; in short, they may lack the “cultural maps” (Wright, 2005) to successfully navigate the prison and its classrooms. They may also inadvertently take on the values and logics of the prison system and contribute to the further oppression of incarcerated students (Ginsburg, 2019).

Notably, several program faculty highlighted that their orientation and training from the prisons emphasized the need for discipline and control, and the potential threat of danger in the classroom—in one case, even comparing students to middle schoolers who require close supervision. Such a focus shifts the purpose of education from learning and development to behavior management, reducing students to security risks and eroding trust between educator and learner even before instruction begins. As Royer et al. (2023) argue, it is critical to consider the countless forms of “prison-initiated discipline” (p. 242) that not only inhibit students’ education and growth, but constrain educators in their ability to provide high-quality, engaging experiences for students. The disciplinary power and control exerted in prison-based educational programs echo long-standing patterns of punitive educational practices in K-12 education, disproportionately impacting marginalized Black, Brown, and low-income students. As is well documented, discipline in these contexts often manifests through surveillance, exclusion, and control, mirroring carceral logics more than developmental support, and entrenching the school-to-prison pipeline (e.g., Gregory et al., 2010; Okonofua & Eberhardt, 2015; U.S. Department of Education Office for Civil Rights, 2016). This resemblance is not coincidental and instead reflects a historical debt that conditions certain populations to expect authority as authoritarian and education as control.

Although far in the minority, a few faculty participants expressed that further training was not necessary because their training as college educators and, for one participant, as an educator in a different prison, sufficiently prepared them for the prison classroom. This logic is also emblematic of historical debt—Scott (2013) cautioned that rationales of this nature reflect a long-held, positivistic approach to education, wherein teachers focus on content and curricular proficiencies while ignoring issues of power and privilege. Ultimately, positioning the lack of comprehensive faculty training in the HEP realm as an exacerbation of historical debt serves to highlight the pervasiveness of what critical education scholar, Love (2019), described as the “teacher education gap” across the educational pipeline. Greater care and thought in the area of faculty training in HEP can increase the potential for mutually humanizing pedagogical experiences in prison.

### 4.2. Educational Experiences in the Programs and Education Debt

The students in the three programs under study, like many postsecondary students in prisons, do not have access to many of the experiences and privileges that are guaranteed to college students on non-carceral campuses (Erzen et al., 2019). For example, our study highlighted that students in the HEP programs did not have sustained and regular interaction with faculty outside of class; regular opportunities for peer learning and collaboration outside of class; access to Internet and academic libraries—and by extension opportunities for independent research; nor a wide range of student support services and resources. Research highlights that there are real implications to not providing these opportunities. For example, abundant empirical research has linked student–faculty interaction—both inside and outside of the classroom—to a host of positive outcomes, including but not limited to academic motivation (Komarraju et al., 2010; Trolian et al., 2016), gains in critical thinking (Y. K. Kim & Sax, 2011; Umbach & Wawrzynski, 2005); enhanced academic self-concept and motivation (Cole, 2011; Y. K. Kim & Sax, 2014; Mayhew et al., 2016), and enhanced professional development and career preparation (Chambliss & Takacs, 2014; Flowers, 2004). Peer learning and collaboration have been shown to enhance retention and persistence (e.g., Braxton et al., 2004; Crisp, 2010); learning gains (e.g., E. Bettinger et al., 2016; Butler-Paisley & Clemetsen, 2019); gains in academic self-concept (Cole, 2011; Y. K. Kim & Sax, 2014); and stronger problem-solving skills (Roscoe & Chi, 2007; Zevallos & Washburn, 2014), to a name a few benefits. Furthermore, research has shown that academic library usage is linked to greater academic achievement and information literacy (e.g., D. Moore et al., 2002; Soria et al., 2015). That students in the three programs have little or no access to student support services from their main campuses (e.g., academic advising, tutoring, Writing Center, counseling and wellness) remains problematic, as research has shown that high-quality student services—particularly integrated services that combine multiple forms of support—enhance academic achievement E. P. Bettinger & Baker, 2014; Sanchez et al., 2006).

The lack of Internet access for students in these programs as well as in most HEP programs across the country not only highlights persistent disparities in higher education in prison but also serves as a poignant example of moral debt. Although it is abundantly clear that all students need to build their digital competency to support their personal, professional, and educational success after incarceration, the prisons in which the programs under study take place and/or the DOC have not figured out a way to provide students with Internet access. Lack of Internet access also prohibits students from directly accessing university library resources, a key component of the postsecondary experience that nobody would find acceptable for a non-incarcerated college student. Even in the best-case scenario of the MU program—where librarians assist students with accessing library resources—faculty noted that the one-week delay in getting resources justifiably caused undue stress on students. Lack of Internet also prohibits students in prison from independently identifying and evaluating information sources and conducting independent research. Numerous studies have documented that generally speaking, undergraduate research can help students build a range of analytical skills, enhance communication skills, build confidence, and develop their ability to work independently (e.g., Jones et al., 2010; Kaul et al., 2016; Lopatto, 2007). What is more, as Castro and Brawn (2017) note, incarcerated students’ “inability to freely access information and to exist in the world as independent thinkers” (p. 103) precludes their participation in any kind of a critical pedagogy in prison. It is worth noting that while these debts have the greatest impact on students, they also disadvantage faculty. As evidenced in this study, college-in-prison instructors must often compensate for the scarcity of resources and services through their own time and labor. Like library access, the lack of dedicated and quiet spaces for study in all but the MU program—and in many prison-based education programs—presents a moral and ethical issue as much as it does a logistical one. On any traditional college campus, the existence of libraries, study rooms, and silent areas is considered a non-negotiable aspect of academic life. These spaces are essential for deep learning and intellectual exploration. To deny these same conditions to incarcerated students undermines the very mission of higher education.

In addition to moral debt, we maintain that an examination of the educational experiences afforded in each program illustrates economic debt. Ladson-Billings (2006) defined economic debt as the historical and contemporary funding disparities in education. Students in the programs represented in this study not only lack direct access to the campus library, the Internet, and support services on campus, but classroom descriptions from study participants also revealed loud and/or cramped classroom spaces, outdated technology, and inadequate resources for teaching and learning. This finding is not simply a reminder that prisons are designed to punish rather than educate—it exposes the underfunding of HEP programs, reflecting a longer history of inequitable spending and distribution of resources for disenfranchised and underserved students (e.g., Bruce et al., 2019; Ladson-Billings, 2007; Milner, 2010). It is well documented that the highest poverty K-12 school districts and districts serving large proportions of students of color receive less funding per pupil than other districts (Education Law Center, 2015). When this historical reality intersects with the criminal legal system, the economic and historical debt is further compounded—often times, educational programming in prison is deemed a worthy investment provided that it reduces recidivism, saves taxpayer money, and decreases prison spending (Castro & Gould, 2018). In other words, it is not a worthy investment in its own right, as something that supports the continued education of incarcerated individuals.

Despite these critiques, the college-in-prison programs in this study also challenge education debt by providing academically challenging experiences, opportunities for community-building, and coursework that reflects student subjectivities and lived experiences. Across programs, faculty indicated high levels of academic challenge in their courses, through incorporation of higher order as well as reflective and integrative thinking. Faculty described course content that, for example, allows students to connect learning to prior experiences or knowledge and evaluate issues from different perspectives. They also described rich class discussions that challenge both students and faculty. Higher education researchers indicate that these types of higher order as well as reflective and integrative thinking foster deep learning and enable students to fully participate in their academic, social, civic, and professional lives (Brierton et al., 2016; Ghanizadeh, 2017); they have also been associated with greater subject matter competence (e.g., Deslauriers et al., 2011; Maskiewicz et al., 2012).

Faculty and staff in the three programs also highlighted the ways in which classroom experiences facilitate community-building. Various HEP scholars and practitioners have described their experiences in similar ways (N. B. D. Moore, 2015; Novek, 2019). They emphasized that the sense of community that emerges in some HEP classrooms is far from a given and can provide rare moments of normalcy and humanity as well as a respite from the routine of prison. In a controlled and often segregated context like a prison, the college classroom offers one space to bring people together in the common pursuit of knowledge and exploration. As J. Davis (2018) described:

I am a prisoner, but in the classroom, I am part of a special community that is dedicated to learning…It is special to be within this harsh prison environment and be able to experience, even momentarily, some semblance of normalcy. There is also a positive culture within that community that is entirely different from the culture in general population. In the classroom space ideas are shared and debated, intellectual growth is fostered, and friendships can transcend prison and the normal prison routine of separation

(p. 3).

McCorkel and DeFina (2019) added that prison higher education enables people who—based on religion, race/ethnicity, age, political ideology, or geographical origin—might not otherwise interact with one another be part of an enriching intellectual community. And as faculty participants in this study noted, the prison classroom provides a community “that isn’t controlled in the way the rest of prison is” and can offer personal and moral support around difficult life circumstances. In this way, HEP and its participants establish and redefine community within the context of the prison in multiple ways.

In addition to community-building, select faculty and staff across programs also emphasized the ways in which course content centers students’ lived experiences and subjectivities. Indeed, Scott (2013) highlighted that for HEP to realize its liberatory potential, it must be guided by students’ subjectivities. Some faculty participants described addressing issues that impact many of the communities from which students originate (e.g., racism, policing, residential segregation) from historical, contemporary, and sociological perspectives. Simpson (2019) reinforced the need for educators to understand students’ life experiences and provide opportunities for “students to take up subjects that concern their communities, rather than relying on abstract theory” (p. 34). Although empirical data on the topic is sparse, various scholars have noted that college-in-prison programs can and should foster students’ abilities to participate in social action and change (Castro et al., 2015; Scott, 2013; Simpson, 2019). In this study, faculty described assignments or projects that asked students to think through particular community or social issues and propose actions to address them. As described previously, the college-in-prison programs in this study simultaneously exacerbate educational inequities and, in community with students, provide important opportunities for intellectual development and human connection.

### 4.3. Limitations

Although this study is one of only a small number of empirical studies investigating the nature and quality of HEP program in the United States, and the only known study focused specifically on program faculty and directors, it is important to highlight key limitations. First, the study only highlights credit-bearing higher education programs in prisons in the southern United States. Incarcerated students in many states also take vocationally oriented coursework towards certificates of completion, and some may access extension courses. These opportunities are important and worth investigating in their own right but were not a focus of this study.

Furthermore, in focusing this research on one region, the extent to which study findings will be transferrable will depend on how similar the historical and contemporary context of the criminal legal system and prison-based education is in other places. As such, the findings may be particularly informative for other states in the Southern US, but perhaps less so for states like California and Washington, which in some cases have had more progressive policies and practices for delivering higher education opportunities in prisons. What is more, given the variation of historical, educational, and political contexts within any given region of the country, it is not necessarily the case that study findings would be directly applicable for other southern states. In an attempt to address this limitation, we provided as much information as possible about the programs to allow others to determine the degree of transferability to their own contexts. Also, although this study examines faculty training as well as the educational experiences afforded to students to consider the quality of HEP programs, “quality” in higher education is conceptualized in the same way, just as there are no universally agreed upon goals of education more generally. Like any study in education that examines quality and practice, this fact should be considered.

Although some exploratory studies have centered the student voice and highlighted the positive impacts of prison education on youth self-esteem and/or personal development (e.g., Carrigan & Maunsell, 2014; Clarke MacMahon & O’Reilly, 2015), the student voice is absent from this research—and it is clearly important to understand student perspectives about the nature and quality of HEP programs. That said, while this is a limitation, it is also important to recognize the extent to which integrating student voices in HEP research comes with a range of ethical and practical considerations with which researchers must grapple (e.g., the extent to which HEP student participants may put themselves at risk by sharing their observations and critiques).

Despite the aforementioned limitations, the present study is a unique contribution to the relatively small body of empirical and critical scholarship on HEP. We contend that it has implications that could meaningfully inform and enhance the practices of the HEP programs under study as well as potentially informing other college-in-prison programs.

### 4.4. Study Implications

As the aim of critical inquiry is not only to interrogate systems of power and oppression, but to foment change (Crotty, 1998), we consider the experiences of our participants as well as broader insights from scholars in the field to generate some recommendations and considerations for practice, policy, and research. With that goal in mind, however, we acknowledge that college-in-prison is a challenging and oftentimes fraught endeavor. The programs in this study, like many other programs, are under-resourced, understaffed, and must navigate the priorities and regulations of prisons and the DOC. As such, we offer the following summary table of recommendations for practice, policy, and research with humility (see Table 2). The recommendations do not reflect an exhaustive list, but we aimed to outline several priorities.

## 5. Conclusions

Perhaps nowhere is the persistent inequality in education that Ladson-Billings (2006) speaks of more apparent than in the prison context. Ultimately, the central tension in examining the nature and quality of the college-in-prison programs in this study is that they at once contribute to, and challenge, the education debt. On the one hand, college-in-prison programs represent a logical conclusion to historical inequities in K-12 education and a fundamentally unjust criminal legal system. Lack of comprehensive faculty training and chronic resource constraints bar college students in prison from accessing the same opportunities as their non-incarcerated peers. On the other hand, the programs in this study challenge the education debt—even though they operate in the brutal and resource-deprived environment of prison, students and faculty co-create rich spaces for exploration, learning, and community-building. In many ways, this has always been the history of education by and for marginalized populations, where under the most oppressive, inhumane, and violent circumstances, such communities have fought to educate themselves (Ladson-Billings, 2006; Love, 2019). In an era of mass incarceration in the United States, there are no lasting and deep disruptions of education debt; HEP, at best, can offer momentary ruptures. Still, it remains no less critical for college-in-prison program faculty, staff, and students to collectively foster academically challenging and mutually humanizing postsecondary educational environments and experiences within prisons.

## Figures and Tables

**Table 1:** Study Participant Demographics by Program.

	Lake (*n* = 4)	Mountainside (*n* = 6)	River State (*n* = 11)
Gender Identity			
Woman	2	3	7
Man	2	3	4
Race/Ethnicity			
White	4	5	9
Asian	0	1	1
Two or more races	0	0	1
Age			
Under 29	0	0	2
30–39	0	1	7
40–49	1	1	2
50–59	2	2	0
60 or older	1	2	0
Highest Education			
Associate’s	1	0	0
Bachelor	1	0	1
Master’s	2	1	5
Ph.D.	0	5	4
Professional ^a^	0	0	1
Median yrs. working in higher education	12.5	15	7
Median yrs. working in prison-based education	6.75	<1	2

^a^ Professional refers to degrees such as M.D., J.D., D.D.S., etc.

**Table 2:** Summary of Recommendations and Considerations.

Primary Area	Recommendation/Consideration	Brief Description of Recommendation
Practice	Collectively develop or refine program mission and goals	Involve faculty, staff, and students in developing—or refining—a shared mission and program goals to guide the work, provide greater alignment among all those involved, and inform and strengthen recruitment and hiring practices.
	Establish and communicate clear policies around language use in programs	Various faculty and staff across programs in this study referred to students on multiple occasions as offenders or inmates. Many members of the broader HEP community—including currently and formerly incarcerated people—have continued to contest the use of dehumanizing terminology to refer to individuals in prison (Castro et al., 2015; Ellis, n.d.). HEP programs must educate faculty and staff about broader debates surrounding terminology and establish well-defined policies around language use so as to avoid the further oppression of incarcerated individuals.
	Develop a faculty handbook	HEP programs can strengthen faculty orientation and preparation through developing a faculty handbook. To assist with this process, staff can consult examples of faculty handbooks in AHEP’s document library, which is freely available on their website (https://www.higheredinprison.org/national-directory/document-library, accessed on 30 June 2025).
	Develop and provide faculty orientation and ongoing training from the program side	It is critical that programs develop their own faculty orientation and training to ensure that a higher education—as opposed to correctional—perspective is provided for faculty and staff. Programs should be aware of the information and messaging that faculty receive from the prisons and be clear with instructors about the program’s philosophical stance and goals. Programs can also help faculty navigate the inevitable tensions that arise between the corrections and education sides as well as the problematic ways in which the two overlap.
	Learn with and from other college-in-prison programs	More than 370 colleges and universities in the US provide postsecondary education in prisons (Royer et al., 2020). HEP programs should engage this community to provide and receive guidance on particular areas of operation. To search for comparable programs, faculty and staff can consult AHEP’s directory (https://www.higheredinprison.org/national-directory/list-view, accessed on 30 June 2025), which lists the sponsoring institution, type of program, program profile with general information, and contact name and information.
	Incorporate more student voice and participation	At minimum, programs should systematically collect course evaluations and provide additional avenues for students to offer feedback about the program as a whole. Programs that have the capacity to do so can consider creating formal mechanisms like a student advisory council to allow greater, institutionalized guidance and participation from current and former students about program planning, administration, and evaluation (Erzen et al., 2019).
	Collaborate with undergraduate and graduate students to increase program capacity	In light of staffing, resource, and time constraints, HEP programs can consider working with undergraduate and graduate students to address program needs. For example, in consultation with HEP experts and as part of an independent study course or research project, a graduate student with an interest in—and in-depth knowledge of—prison higher education could assist with creating a faculty handbook and/or developing faculty training materials.
Policy	Formalize faculty recruitment and hiring procedures	According to study participants, faculty recruitment and hiring is currently an informal and inconsistent process. To ensure that faculty are aligned with program mission, philosophy, and goals—and that they are provided with clear expectations around faculty training and other responsibilities—it is important that programs formalize their recruitment and hiring procedures and consult other programs that already have these procedures in place to assist with the process.
	Require faculty orientation	To formalize faculty onboarding, it is recommended that programs require all new faculty to attend an orientation, in which facilitators walk through the Faculty Handbook to review program mission, history, background on the prisons, general information about the students; and program and prison policies.
	Require faculty training (beyond training associated with corrections or prison policy)	To ensure ongoing professional development for HEP faculty, programs should consider providing trainings throughout the year. Given limited time and resources, programs need not concern themselves with putting on formal events, so much as providing meaningful and ongoing opportunities for discussion and reflection, resource sharing, and problem solving.
	Consider opportunities for students to access Internet in an appropriate manner	Though few, there are examples of prisons that have set up closed Internet access—the prisons in the programs under study and the DOC can seek out and consult other facilities about how they have successfully implemented these resources.
	Consider opportunities for students to have dedicated study space	This need not be a new space—the MU study hall is in the same room where students have class; they simply are provided with additional designated time for out-of-class work and collaboration.
	Hire staff (or more staff) with education backgrounds	To ensure that college-in-prison programs—and educational programming in general—are well-supported, it is recommended that the prisons and DOC hire staff with explicit training in education. The more the prisons have staff who truly understand education, the greater the possibility that students will gain access to the appropriate and needed support and resources.
Research	Conduct research incorporating the student perspective	To fully understand what kind of educational experiences college-in-prison programs provide, it is necessary to incorporate the student perspective. At the same time, to embark on research that involves disenfranchised populations such as incarcerated students, it is of utmost importance that researchers first educate themselves about the problematic history of research in and on prisons, and design studies with insight from experienced practitioners and scholars as well as those with relevant lived experiences.
	Conduct in-depth research on particular HEP program practices	For example, research can highlight those programs that have rigorous and thoughtful faculty training to provide models for other programs. What do these programs do? What materials and resources do they use? How, if at all, have they collaborated with students?
	Conduct further research on the experiences and perspectives of HEP faculty and staff	If we want to see college-in-prison programming grow and thrive, we must better understand and support multiple facets of the faculty experience. One study participant, for example, noted that research is needed on the emotional aspects and toll of teaching in prison. There are numerous other research questions that can be asked to better understand the experiences and perspectives of HEP faculty and staff.

## Data Availability

The original contributions presented in this study are included in the article. Further inquiries can be directed to the corresponding author.

## Notes

1. For the purposes of this manuscript—taking into consideration overall concision, readability, and efforts to remain consistent with existing critical scholarship on this topic—we sometimes use phrasing that does not adhere to person-first language recommendations. However, we fully value and acknowledge the importance of person-first language, and in the context of our study, any phrasing such as “incarcerated individuals or students” should be interpreted as “individuals or students who are incarcerated.”

2. Throughout this document, college-in-prison program, prison-based college program, prison-based higher education program, prison-based postsecondary education program, and higher education in prison (HEP) are used interchangeably to refer to college programming in prison. Also, unless otherwise specified, reference to national data, federal policy, or general trends for college-in-prison programming is referring to circumstances within the United States of America.

3. As author and educator, Kilgore (2011), who also served as a peer instructor within various prison education programs while incarcerated in California notes, prisons are full of “invisible educators,” who support the learning and educational trajectory of incarcerated students.

## Abbreviations

The following abbreviations are used in this manuscript:

HEP	Higher Education in Prison
DOC	Department of Corrections
US	United States (of America)
